# Countering a Drone in a 3D Space: Analyzing Deep Reinforcement Learning Methods

**DOI:** 10.3390/s22228863

**Published:** 2022-11-16

**Authors:** Ender Çetin, Cristina Barrado, Enric Pastor

**Affiliations:** Computer Architecture Department, UPC BarcelonaTECH, Esteve Terrades 7, 08860 Castelldefels, Spain

**Keywords:** counter drones, UAV, deep reinforcement learning, DQN, dueling DDQN, DQfD, prioritized experience replay, transfer learning, object detection, Yolo

## Abstract

Unmanned aerial vehicles (UAV), also known as drones have been used for a variety of reasons and the commercial drone market growth is expected to reach remarkable levels in the near future. However, some drone users can mistakenly or intentionally fly into flight paths at major airports, flying too close to commercial aircraft or invading people’s privacy. In order to prevent these unwanted events, counter-drone technology is needed to eliminate threats from drones and hopefully they can be integrated into the skies safely. There are various counter-drone methods available in the industry. However, a counter-drone system supported by an artificial intelligence (AI) method can be an efficient way to fight against drones instead of human intervention. In this paper, a deep reinforcement learning (DRL) method has been proposed to counter a drone in a 3D space by using another drone. In a 2D space it is already shown that the deep reinforcement learning method is an effective way to counter a drone. However, countering a drone in a 3D space with another drone is a very challenging task considering the time required to train and avoid obstacles at the same time. A Deep Q-Network (DQN) algorithm with dueling network architecture and prioritized experience replay is presented to catch another drone in the environment provided by an Airsim simulator. The models have been trained and tested with different scenarios to analyze the learning progress of the drone. Experiences from previous training are also transferred before starting a new training by pre-processing the previous experiences and eliminating those considered as bad experiences. The results show that the best models are obtained with transfer learning and the drone learning progress has been increased dramatically. Additionally, an algorithm which combines imitation learning and reinforcement learning is implemented to catch the target drone. In this algorithm, called deep q-learning from demonstrations (DQfD), expert demonstrations data and self-generated data by the agent are sampled and the agent continues learning without overwriting the demonstration data. The main advantage of this algorithm is to accelerate the learning process even if there is a small amount of demonstration data.

## 1. Introduction

The drone industry improved itself over the years and the drone market is growing dramatically. According to Bloomberg [1], commercial drone market is estimated to be worth United States Dollar (USD) 6.5 Billion in 2022 and is forecast to a readjusted size of USD 34.5 Billion by 2028 with a Compound Annual Growth Rate (CAGR) of 32.0% during the review period. Drones are used by professionals and hobbyists for different purposes such as delivery, wildlife monitoring, reconnaissance, inspection and surveillance. The United States Federal Aviation Administration (FAA) published a report [2] indicating that 865,505 drones are registered. In this report it is stated that 314,689 are commercial drones and 538,172 are recreational drones or for hobby use. These numbers are quickly increasing all over the world.

Artificial intelligence (AI) has been utilized in different purposes to support Unmanned air vehicles (UAV). For example, a drone supported with AI can navigate in an unknown environment by detecting and avoiding the obstacles by using object detection algorithms. Moreover, a drone can deliver medicine or any kind of materials by operating autonomously using an AI method. Reinforcement learning (RL) which is an AI method based on trial and error experiences, is also used in drones in different scenarios. Drones supported with RL can operate autonomously to deliver goods, navigate in an environment, or even in drone racing tournaments where drones race against human pilots. Reinforcement learning methods showed promising results in many areas such as gaming which requires a lot of experiences to achieve successful results. This shows that a drone supported with RL can be used to counter drones in an effective way. Fighting against unknown and malicious drones can be very accurate and efficient by implementing an AI method in counter-drone technology. AI methods can speed up the time to engage with the target compared to other methods based on human intervention. A drone with AI can identify and classify the target with a high precision. It is also possible to prevent a false interdiction with the targeted object by using an AI. Countering a drone in 2D space can be an easy way since the drone and the target move without changing altitude. However, if the target changes an altitude and moves in 3D space, which is highly expected in real world, an AI method such as reinforcement learning can be an efficient method thanks to perception and interpretation of environment by RL agent drone since RL models can learn by interacting in an environment by trial and error experiences. This is an important advantage to be used against drones in 3D space.

## 2. Contributions and Related Work

### 2.1. Counter-Drone Systems

The increasing use of commercially available drones and their growing capabilities are posing a threat to the safety of the skies if they are misused. In order to eliminate threats to public security and privacy because of these misused drones, counter-drone solutions have been proposed by research using different methods and tools. Security professionals have also used different techniques to eliminate unwanted drones by using a laser gun, jamming, throwing a net to capture a drone, a water projector, and an animal such as an eagle to hunt down a drone.

In this paper, detecting the target location is not considered and the related data are already available thanks to the flight simulation used to train and test the DRL methods. More details on detecting drones can be seen in a survey by Chiper et al. [3]. In this survey, the different drone detection and defense systems based on different types of methods which were proposed in the literature are presented. In the real world, the target position can be detected in many different sensing technologies such as radar [4,5], acoustical [6,7], radio frequency (RF) [8], optical [9], lidar [10], or a deep learning-based solution [11,12].

Although there are many counter-drone solutions available in the literature, each solution targets special cases. In a survey [13], the limitations of available counter-drone technologies and the advantages of using them are explained in detail. In this study, it is stated that the threats coming from drones in airports are not easy to deal with. However, methods such as geofencing, multiple radars with different detection ranges and a combination of radio-frequency sensors with visual detection sensors can be implemented to defend airports against unwanted drones. It is also highlighted that airfield operators must remain within the law when using disruptive technologies, and the risks to the wider community should be fully assessed and understood.

Researchers Watkins et al. proposed a blueprint [14] which offers a design for a novel autonomous counter drone tool based on the weaponization of “hard-to-patch” vulnerabilities. The paper highlights the problem of privacy violation due to drones and presents a counter-drone tool which breaks the drone’s autonomy code.

In another study [15], it is presented that the system developed can extract target UAV trajectory which is enough to intercept an intruder drone. The research states that with a priori knowledge of the shape of the target trajectory, they managed to track and intercept an intruding drone 30% faster than their sentry vehicle in more than half of the software in the loop (SITL) experiments conducted. The system is also tested in an outdoor unstructured environment and the drone successfully intercepts in 9 out of 12 experiments.

### 2.2. Reinforcement Learning

AI methods such as reinforcement learning (RL) have been used in drones for a variety of reasons. Reinforcement learning is an approach to AI based on trial-and-error experiences. Drones equipped with RL can navigate in an environment with obstacles without crashing on them. Researchers have been proposing their studies in the area of reinforcement learning, and drones and their main focus is on navigating drones in an unknown environment, avoiding obstacles, and chasing drones. Reinforcement learning is also used in [16] to focus on automated anti-collision systems. In this study, it is stated that training Reinforcement Learning agents can deflect a drone equipped with an automated anti-collision system. The effectiveness of reinforcement learning in finding security holes for the autonomous systems is also highlighted. More3over, in a study by Lee H. [17], tracking and capturing an invading drone using a vision-based drone to defend it is presented. Firstly, researchers developed a deep learning-based detection algorithm which applied to detect a drone and estimate its position. Secondly, a deep reinforcement learning algorithm is introduced to find the optimal behavior to track a drone. Moreover, Akhloufi et al. [18] proposed deep reinforcement learning to predict the actions to be applied to the follower UAV to keep track of the target UAV. Furthermore, supervised learning is applied by using a large dataset of drone images. Another example is to predict the position of the target drone using a deep object detector and a search area proposal. Reinforcement learning can be combined with another deep learning method called imitation learning. For example, a deep reinforcement learning method is proposed in [19] to navigate an UAV in an unknown environment using demonstration data. Researchers presented that expert demonstrations can speed up the training process and both the policy and Q-value network are pre-trained in the imitation phase. Simulation results show that UAV can avoid obstacles in an unknown 3D environment.

The first and more significant contribution is the novel filtering algorithm applied during transfer learning. This consists of pre-processing the previous experiences and eliminating those considered as bad experiences. Previously, we presented a counter-drone solution [20], a drone equipped with an AI method deep reinforcement learning double deep Q-network (DDQN) to counter a drone in a 2D space. The drone learns to navigate in a geo-fenced environment and heads towards the target drone. However, the actions are only in a 2D space such as moving forward and yawing left and right. In other words, the target drone is assumed to be at the same altitude as the learner drone which is trying to catch the target. However, in this research, a deep reinforcement learning method with dueling network and prioritized experience replay have been proposed to counter a drone in a 3D space and the experiences are loaded from the previous training by filtering the experiences. The second important contribution is that a DRL model deals with the counter-drone challenge in a 3D space. In this paper, a future work from our previous research, a very challenging 3D space, is addressed and a deep reinforcement learning method supported by a state-of-the-art object detection algorithm [21], with a drone detection model proposed so that the drone can catch the target drone in a 3D space. The learner drone is not only moving in a 2D space but also changing altitudes to eliminate the target drone. Critical part of the counter-drone systems to eliminate the target such as the time to interact with the target and the actions which the drone can use are considered carefully. A detailed explanation of the drone detection model used in this paper is provided in our previous research [11] which proposes a drone detection model trained by using different kinds of images of drones to obtain a more robust drone detector. The last and relevant contribution is the explainability of deep reinforcement learning. The figures which represent the rewards, drone locations, crash positions and the action distribution during training and testing are analyzed and compared with different scenarios and parameters. In other words, the agent behavior is observed and the modifications are done accordingly in training and testing sessions.

## 3. Materials and Methods

In this section, the tools used to train and test deep reinforcement learning algorithms are explained.

### 3.1. Tools

Python is used to train and test DRL algorithms. OpenAI-Gym [22] is a toolkit to simulate reinforcement learning algorithms. It is an open-source interface and is compatible with neural network tools such as Tensorflow [23]. Keras-RL [24], which includes state-of-the art deep reinforcement learning algorithms, is used for integrating the deep learning Keras library. Keras-RL can work with OpenAI-Gym and it is easy to define the developer’s own callbacks. In this paper, Keras-RL callbacks and the functions are edited to use a prioritized experience replay. To train and test the reinforcement learning algorithms, an Airsim [25] simulator is used. Airsim has many capabilities for AI research and development such as deep learning, computer vision and reinforcement learning.

### 3.2. DQN, Double-DQN and Dueling Network Architecture

**Figure 1:**
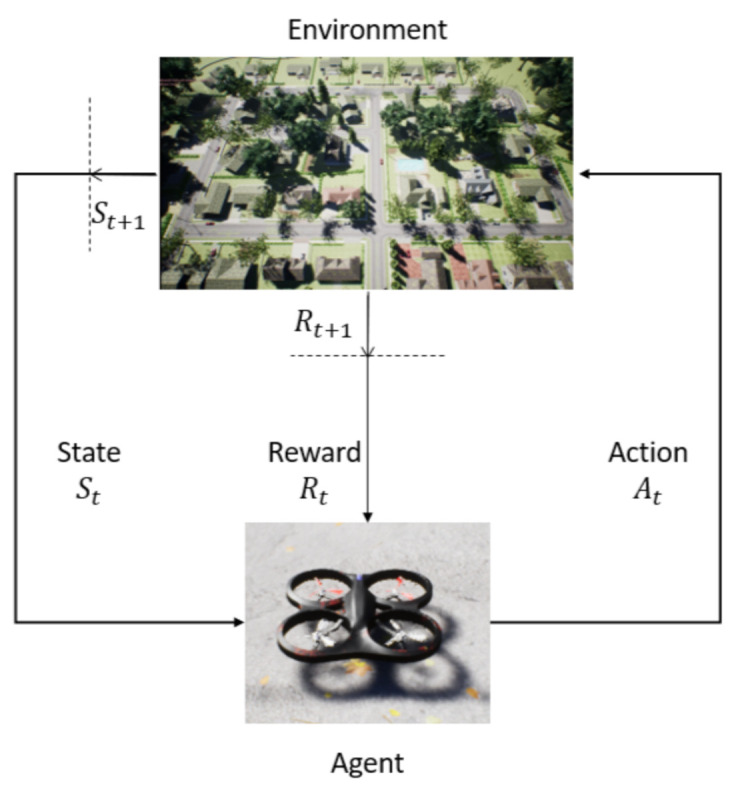
The agent-environment interaction in reinforcement learning.

Reinforcement learning is explained in Section 2.2. RL is an approach to AI inspired by a human’s way of learning, similar to what a baby experiences when learning how to walk. In RL, agent makes a decision and takes an action. The agent interacts with the environment. The environment provides states, which is information about the current status of the agent. Each action updates the environment and its state. Finally, a reward is submitted by the environment informing about the benefit of using the action in that moment. The objective of the agent is to maximize the final reward value. The interaction between the agent and the environment is shown in Figure 1. State is represented as $S_{t}$ and the State Space is represented as *S*. The interaction between the agent and the environment is in discrete time steps *t*. Action and Action Space are represented as $A_{t}$ and $A(S_{t})$ respectively. Reward values are updated in each time, $R_{t+1}$, and a new state becomes $S_{t+1}$.

In RL, states are mapped to the probability of the possible actions in each time step *t* and this is called policy. The policy is chosen to maximize the cumulative reward over time shown in Equation (1). This means maximizing not the immediate rewards $R_{t+1}$, but the cumulative reward over time, called return $G_{t}$. The fundamental concepts for RL are explained in detail in [26].

(1) $$G_{t}≐R_{t+1}+\gamma R_{t+2}+\gamma^{2}R_{t+3}+...=\sum_{k=0}^{\infty }\gamma^{k}R_{t+k+1},$$

where γ∈[0,1] is the discount factor. The discount factor γ determines the importance of future rewards. A factor of 0 will make the agent short-sighted by only considering current rewards, while a factor approaching 1 will make it strive for a long-term high reward.

The DQN method is published by DeepMind [27] and the main goal of DQN is to use a deep convolutional neural network to approximate the optimal action-value function. DQN provides updated action values and target values iteratively. Moreover, it offers an experience replay which randomizes the data and improves the data distribution. Researchers Mnih et al. [27] presented that DRL algorithms can beat a human performance level in video and board games by implementing a double deep Q-network (DDQN) [28], an extension of the deep Q-network (DQN) implementation [29]. The double-DQN (DDQN) algorithm remains the same as the original DQN, except replacing the target of the estimated return as defined as the DQN target. In DDQN, only one estimate is updated per step in a random selection, but two estimates are learned. The memory requirements are doubled but the computational effort made at each step remains the same. The DDQN algorithm is used previously in [20] and it shows promising results in a 2D space when countering a drone. The overall goal of DQN is to use a deep convolutional neural network to approximate the optimal action-value function, defined as:

(2) $$\theta_{\pi }\left(s,a\right)=\max_{\pi }E\left[r_{t}+\gamma r_{t+1}+\gamma^{2}r_{t+2}+\cdots |s_{t}=s,a_{t}=a,\pi \right]$$

The standard Q-learning update for network parameters θ after taking action $A_{t}$ in state $S_{t}$ and observing the immediate reward $R_{t+1}$ and resulting state $S_{t+1}$ is:

(3) $$\theta =\theta_{t}+\alpha \left[y_{t}^{Q}-Q\left(S_{t},A_{t};\theta_{t}\right)\right]\nabla_{\theta_{t}}Q\left(S_{t},A_{t};\theta_{t}\right),$$

where the estimated return as defined as Q-target $y_{t}^{Q}$:

(4) $$y_{t}^{Q}=R_{t+1}+\gamma \max_{a}Q\left(S_{t+1},a;\theta \right)$$

This update resembles a stochastic gradient descent, updating the current value $Q(S_{t},A_{t};\theta_{t})$ over the Temporal Difference (TD) error towards a target value $y_{t}^{Q}$.

**Figure 2:**
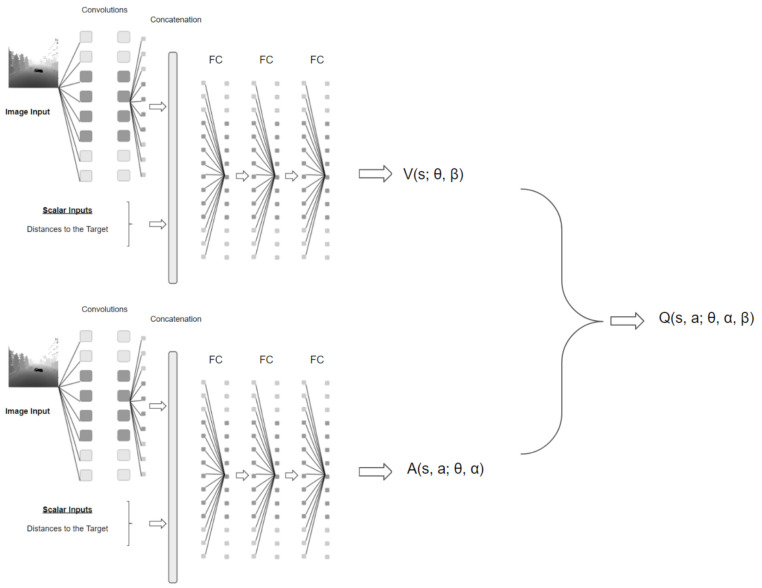
Agent Dueling Architecture.

However, in the dueling network architecture presented in [30] there is no need to estimate the value of each action choice as it is calculated in DQN and Double-DQN. Instead of following the convolutional layers with a single sequence of fully connected layers, the dueling network has two new streams. One of the streams estimates state-value V(s;θ,β) and the other stream estimates the advantage for each action and output an |A| dimensional vector A(s,a;θ,α). θ is the parameters of the convolutional layers, while α and β are the parameters of the two streams of fully-connected layers. The lower layers of the dueling network are as in the original DQN. Finally, the two streams are combined to produce a single output Q function shown in Equation (5) as it is done in DQN [27]. The agent dueling architecture can be seen in Figure 2.

(5) θ(s,a;θ,α,β)=V(s;θ,β)+A(s,a;θ,α)

The advantage of using dueling architecture is that the agent can learn which states are more valuable without learning each action at each state. In other words, there is no need to calculate the value of each action at that state value if the state is not good.

### 3.3. Prioritized Experience Replay

Prioritized Experience Replay (PER) is introduced by Schaul, T. et al. in [31] to make the agent learn faster. Previously, experiences are sampled uniformly from a replay memory. In other words, the transitions are replayed without considering their significance. However, PER prioritized the experiences and important transitions are replayed more frequently. In this way, the agent learns efficiently.

### 3.4. Drone Detection by State-of-the-Art Object Detection Model—EfficientNet

In this paper, EfficientNet-B0, a sub version of EfficientNet [21], is used to detect drones. EfficientNet is a popular state-of-the-art object detection model thanks to its accuracy and efficiency. EfficientNet-B0 is adapted for small-size objects. A detailed explanation of our drone detection model is provided in our previous research [11].

### 3.5. DRL Model

The deep reinforcement learning model in this study was constructed by using dueling network architecture and trained with DDQN including prioritized experience replay. The DRL model is constructed by concatenating an image state and scalar inputs such as distances to the target. The image is an input of a convolutional neural network (CNN), followed by a flatten layer and then a concatenation layer joints the flatten output of the CNN with scalar inputs. Figure 2 shows the neural network model representation including dueling architecture. Details on the environment, states, actions and rewards are presented in the following sections.

#### 3.5.1. Environment

Airsim simulation provides many environments available in Unreal Engine [32] for AI research and development. The urban neighborhood is chosen to counter a drone because of the similarity in real-life experiences such as a high number of drones in urban areas.

#### 3.5.2. States

Agent states consist of images and scalar input values which are concatenated later. However, different image states are used in two different DRL models:

- Image State with Drone Detection

**Figure 3:**
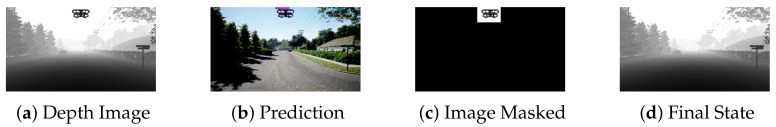
Drone Detection and Image Processing.

Depth image, 84 × 84 pixels, and scene image, 256 × 144 pixels, are captured by using a drone onboard camera. The predicted image seen in Figure 3b is processed by a drone detection model to create bounding boxes when the target drone is detected on the image.
  The depth image seen in Figure 3a is used in the DRL model for detecting obstacles. After processing the images, the bounding box region in the depth image is filled with the color white and circles like a target in a game of darts are created inside the white bounding box region. The final image can be seen in Figure 3d.

- Image State without Drone Detection

**Figure 4:**
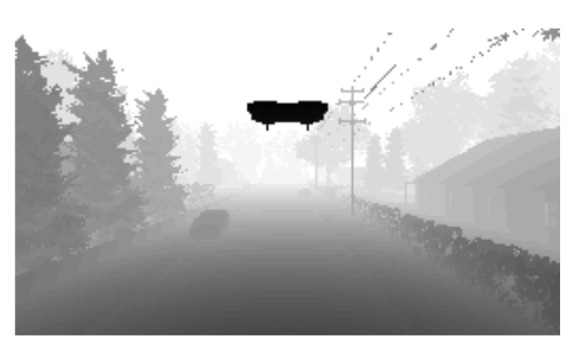
Depth Image.

**Figure 5:**
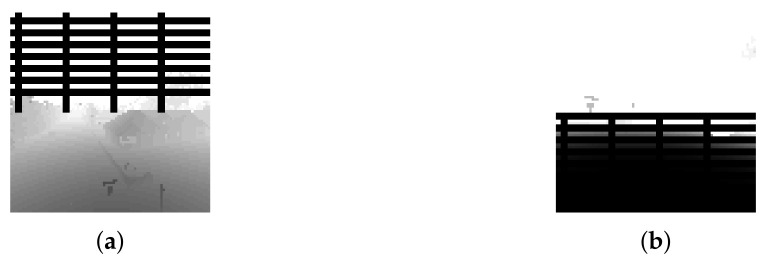
Fences in Image State. (**a**) Grid on Top of the Image. (**b**) Grid on the Bottom of the Image.

The depth image seen in Figure 4, with 256 × 144 pixels captured continuously. This image is the default size that Airsim can output.
  In addition, the grid is drawn on the image if the drone comes closer to the geo-fence limits in all directions. The grids start to be drawn on the image when the distance between the drone and the geo-fence limits lower than or equal to 1 m. The thickness of the grid increases as the drone moves towards the geo-fence limits. An example of the grid image is shown in Figure 5. In Figure 5a, the grid is drawn if the agent is closer to the geo-fences on TOP. On the other hand, in Figure 5b, the grid is drawn on the bottom of the image if the agent is closer to the ground.

- Scalar Inputs

Scalar inputs contain the agent’s distances to the goal in x, y and z directions and the Euclidean distance $d_{x}d_{y}d_{z}d_{t}$.

#### 3.5.3. Actions

**Figure 6:**
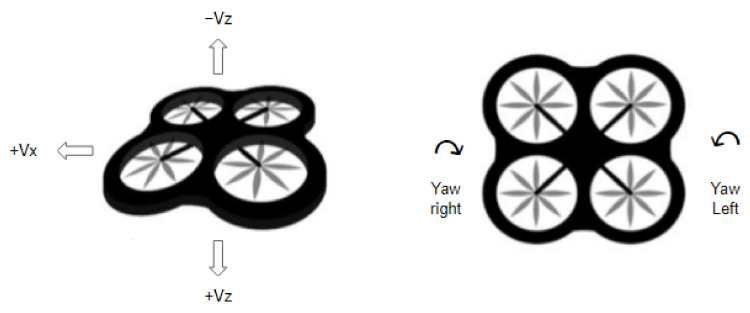
Agent Actions.

The agent can take five different actions such as moving forward, yawing left and right, and going up and down. The actions are represented in detail in Table 1 and in Figure 6.

#### 3.5.4. Rewards

The reward function includes incremental rewards which penalize the agent during the episode and the reward giving a successful episode. In addition, an intermediate step reward is added: ΔDistance which represents the change of distance to the target between the current step and the previous step. In this paper, collision penalization for colliding with any obstacle in the environment is not implemented. The reward function is shown in Table 2.

## 4. Training and Test Results

**Figure 7:**
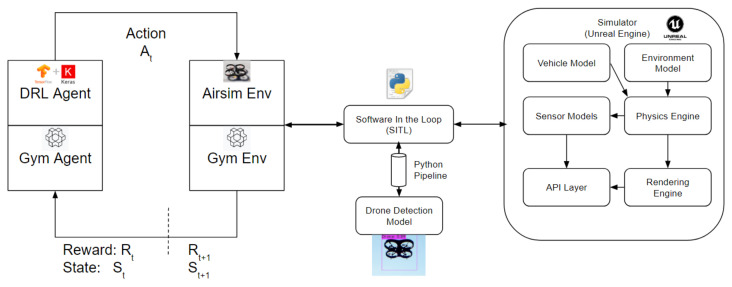
Experimental Setup.

In this section training and test results are presented. Models are trained on a desktop PC with NVIDIA GeForce RTX 3060 Ti with 8 GB VRAM graphics co-processor. In Figure 7, core components of the experimental setup and the interactions between the DRL tools such as Tensorflow, Keras and OpenAI Gym, drone detection model via python pipe which accomplishes the parallel processing and the simulation. The linear Epsilon-greedy policy is applied during the training. Different training steps and the annealed part of the training section are implemented to train DRL models. In addition, the models are also trained by loading experiences from another training. In other words, time to train can be different in different models. In general, full training with 75,000 steps can take approximately 48 h. A summary of the models is presented in Table 3 and Hyperparameters of the training and tests are presented in Table A1. The models are trained by implementing different scenarios such as different target drone locations, teleportation and random heading at the beginning of each episode during training. In addition, some models have been trained by implementing transfer learning and using different network architectures such as a dueling network. Different annealing sections and the total training times are also investigated and shown in Table 3.

**Figure 8:**
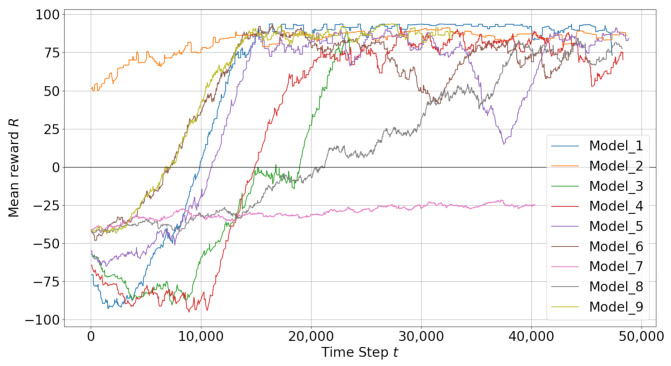
Training Results ALL Models.

Mean rewards of DRL models are presented together in Figure 8. It is seen that only model-2 has positive mean rewards at the beginning and the rest of the training thanks to transfer learning by loading experiences from one of the previous trainings. On the other hand, it can be seen in this figure that one of the models is very slow and does not reach a positive mean reward in training while the other models reach positive mean rewards after some time in training.

In Table 4 maximum, minimum and average cumulative rewards of DRL models are presented. Success rates during training are also shown. Model-1 and model-2 have the maximum success rates and maximum average cumulative results. Model-3 has the minimum success rate with one of the lowest total episode numbers.

### 4.1. Best Models

**Figure 9:**
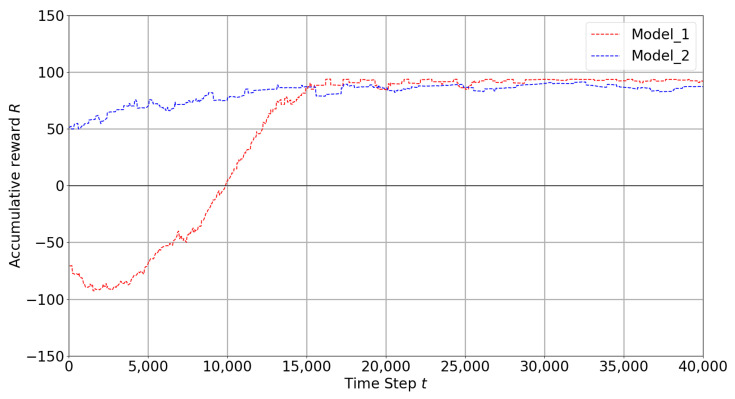
Best Models.

**Figure A1:**
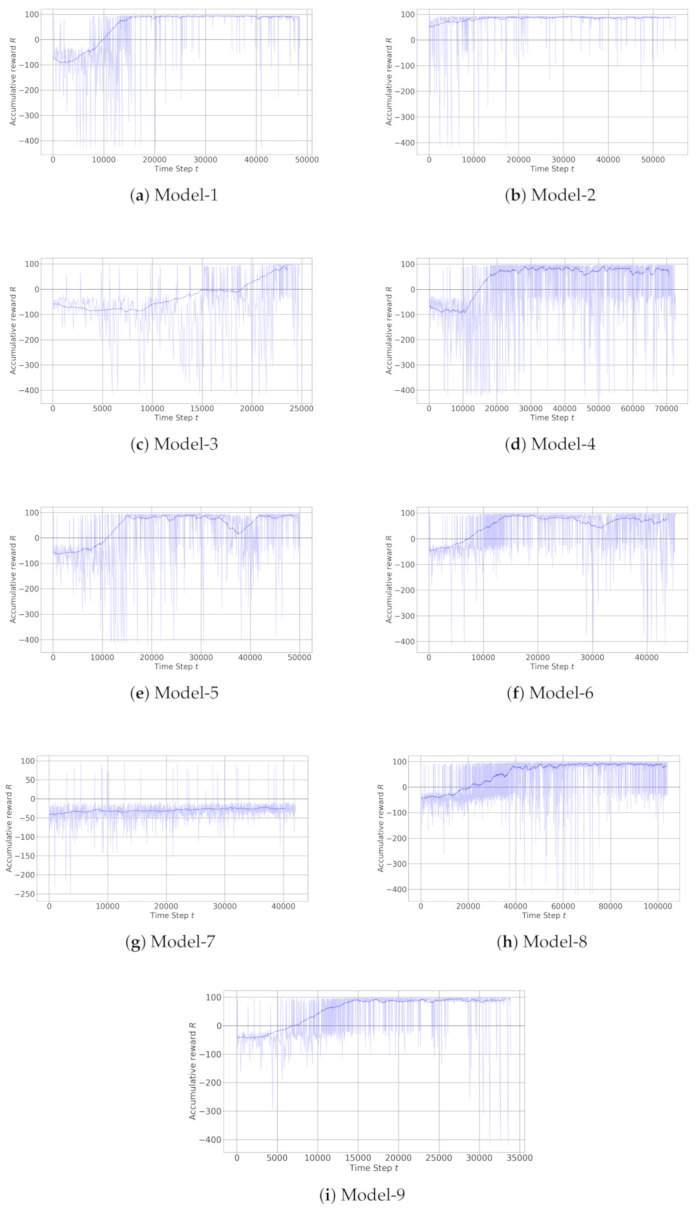
Training Results.

Best models are chosen according to training and test performances. If the training has more successful episodes with less crashes and it is stable during the training, the model is considered to be a good model. All models are presented in detail in Figure A1. Model-1 and Model-2 are selected as best models and the mean rewards are compared and presented in Figure 9. In Table 3, model-1 and model-2 are already described in detail. Although both models have dueling network architecture and prioritized experience replay, model-1 has no drone detection model and no transfer learning. On the other hand, model-2 includes a drone detection model running and the experiences from previous training are transferred. As is seen in Figure 9, model-2 starts the training with positive rewards and reaches its maximum levels in a short time. It is seen that transferring experiences from a previous training speeds up the learning process. However, model-1 starts the training from scratch but can reach the high rewards like model-2, whereas model-1 has more crashes at the beginning of the training.

### 4.2. Analysis of Models

**Figure 10:**
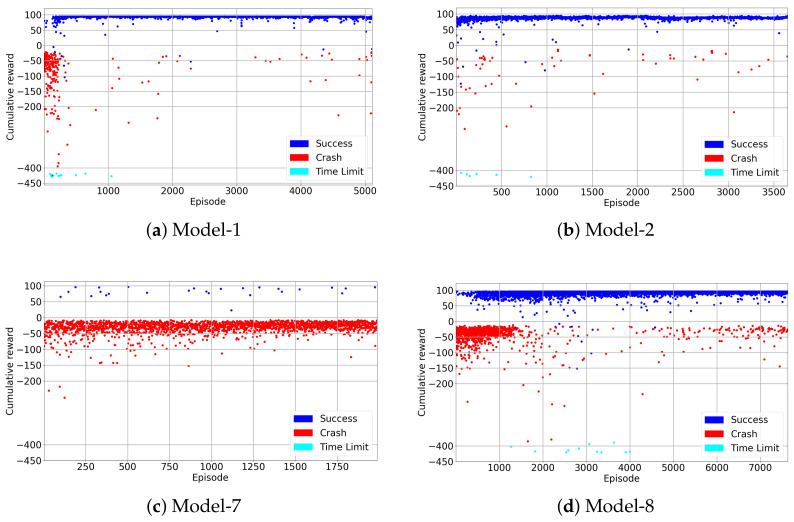
Training Results.

**Figure A3:**
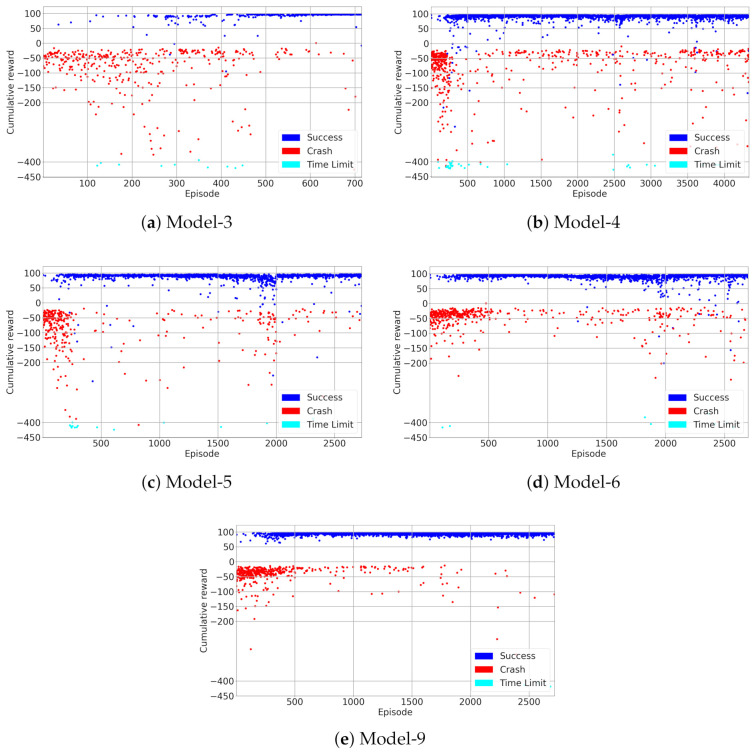
Alternative Training Results.

In this section, the training results of best models are compared with models which were not successful in training. Worst models such as model-7 and model-8 are compared with the best models described in Section 4.1. Model-8 has trained without using transfer learning and has a longer annealing part. Model-8’s training result is shown in Figure 10d. In this figure, the red, blue and cyan colors represent an episode in which the learner drone crashed in the environment, an episode in which the target is caught and the time limit respectively. It is seen that model-7 and model-8 are outperformed by model-1 and model-2. Model-8 is created by starting each episode with random heading without teleportation during training and has more crashes during the training than model-1 an model-2. The training results for model-1 and model-2 are shown in Figure 10a,b respectively. The maximum reward that model-8 can achieve is similar to the other models, but it has many crashes even after the annealing section which ends after 50,000 steps. However, model-7 is the worst model which failed to catch the target during the training, and the training result is presented in Figure 10c. This model is trained with a different scenario such as teleportation and random heading starting at the beginning of each episode, and it has a very long annealing part. The main purpose was to increase exploration by teleporting around the environment, but it was not sufficient for the drone to learn to catch the target. Training results for all DRL models can be seen in Figure A1 and Figure A3.

**Figure 11:**
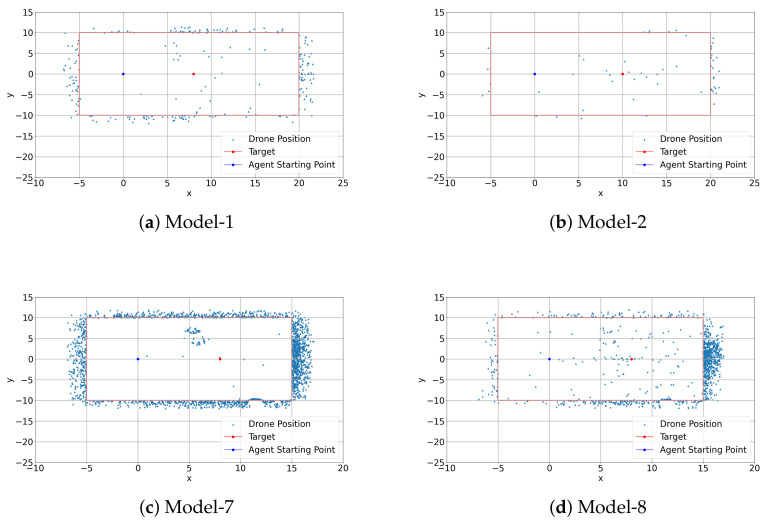
Crash positions.

**Figure A4:**
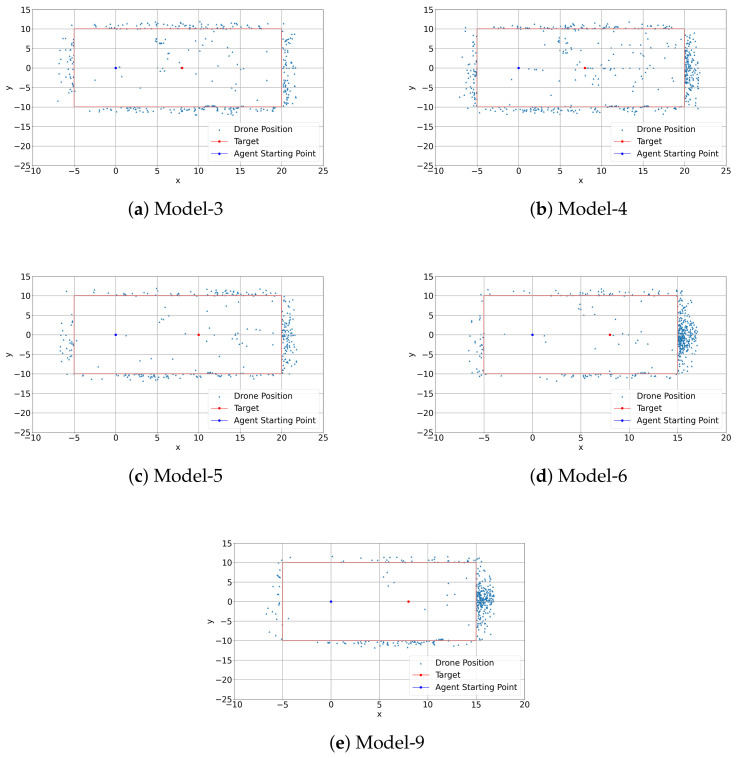
Crashed Episodes.

Additionally, the crashed episodes are analyzed by checking the drone crash locations in the environment. Figure 11 shows crash positions in the environment in x-y directions for four different models. Red rectangle lines represent the geofences in the environment in x-y directions. It is clearly seen that model-7 and model-8 crash a lot of times on the right side of the geofenced location in × direction. However, among all the models, model-2 has minimum crashes. This can also be seen in Figure A4. Model-1 performs better than model-7 and model-8 but not as well as model-2. Moreover, model-7 crashes on each side of the geofenced area. The long annealing part also contributes to this situation because the random behavior is high in the annealing part and the learner drone tries to explore more in the environment, but even after a long time, there are no improvements in this model.

**Figure 12:**
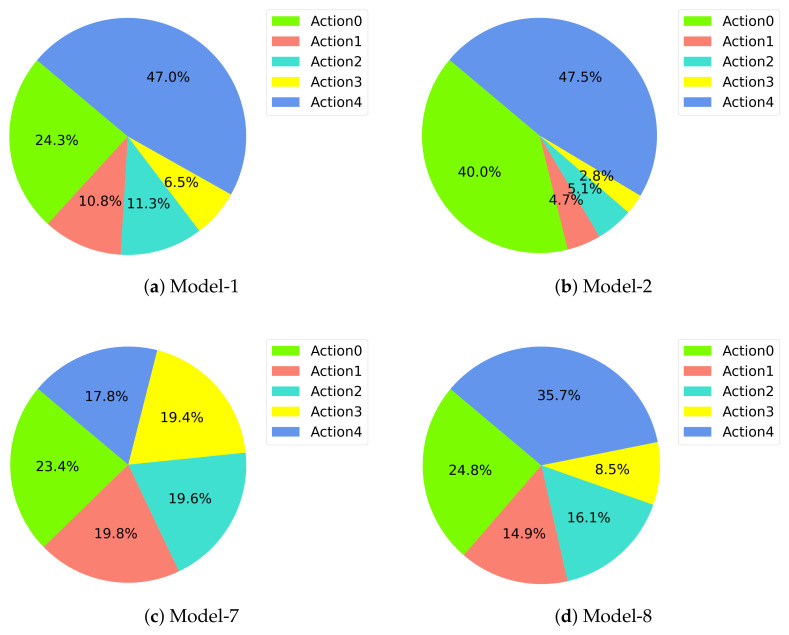
Action Frequencies.

**Figure A5:**
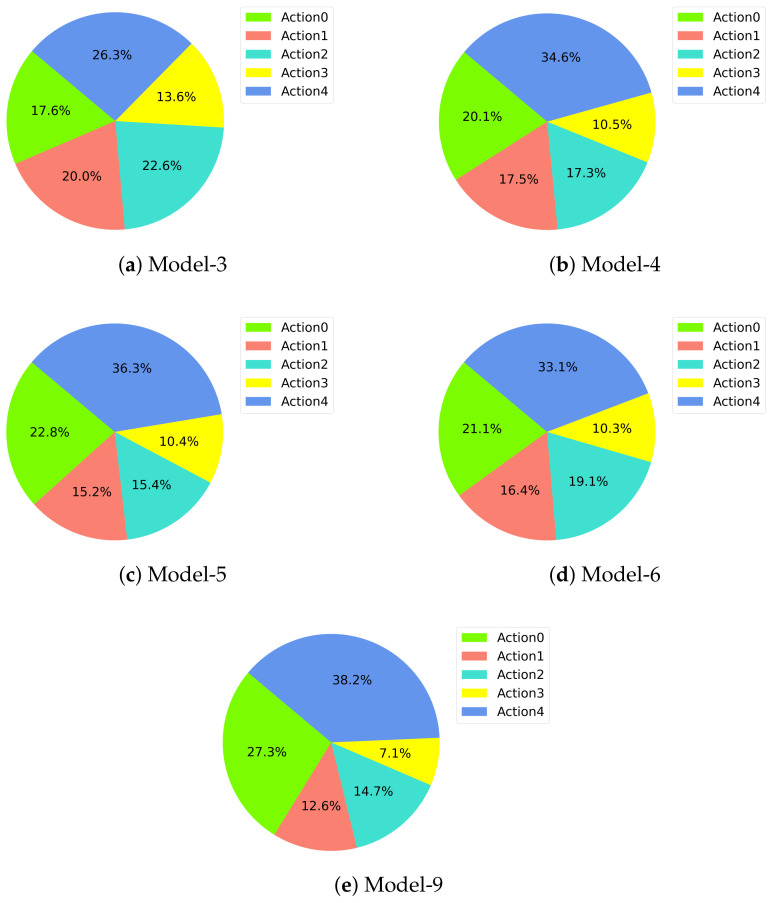
Actions Frequency.

Actions for these four models are also presented in Figure 12. Expected behavior is that the drone should go up and move forward since the target is in front of the learner drone and the vertical distance is 1 m. Model-1 and model-2 perform as expected but model-1 spends more time on turning left and right. Model-2’s performance shows a better result and it spends less time on turning but focuses on going up and moving forward. Model-8 fails to do the expected behavior and sometimes spends a lot of time finding the target. However, model-7’s actions show no learning at all. The actions the drone uses in this model are almost distributed equally among the five actions and the drone has no idea where the target is and where it should move to catch it. All the actions that the DRL models used can be seen in Figure A5.

### 4.3. Test Results

**Figure 13:**
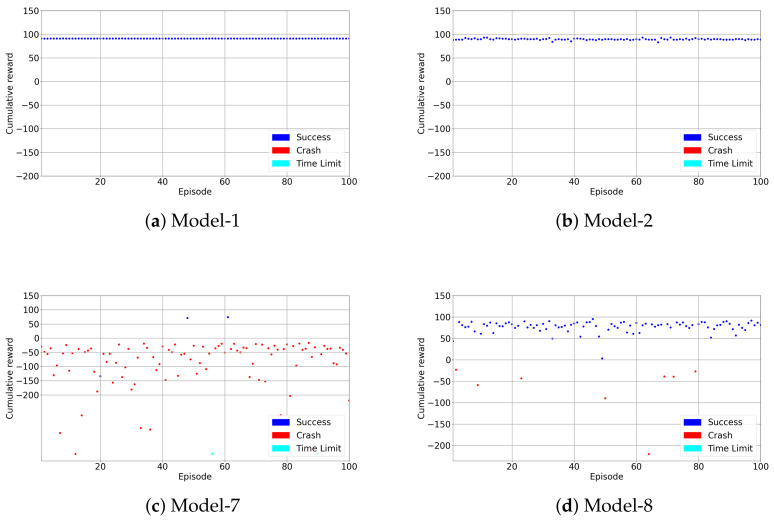
Test Results.

After training the DRL models described in Table 3, the models are tested in the environment with the best checkpoint weights obtained during the training. The models presented in Figure 10 which includes best and worst models are tested and presented in Figure 13. The test results are shown in Table 5. In this table, average cumulative rewards, minimum and maximum rewards are compared. The success rates represent on how many episodes the learner drone catches the target drone in a test out of 100 episodes. In addition, the average steps in each episode in tests are also presented. Model-1 and model-2 show the best performance as expected since they are selected as the best models and shown in Section 4.1. Average cumulative rewards are 90.86 and 89.38, the highest for model-1 and model-2 respectively. The highest minimum cumulative rewards show the precision of these models. Model-1 and model-2 spend less time to catch the target with average time steps 8.98 and 11.92 respectively. However, model-7 and model-8 are not as successful as expected. Although model-8 has a better success rate (92%), it has higher average steps compared to the best models, and model-7 fails to catch the target drone.

## 5. Discussion

**Figure 14:**
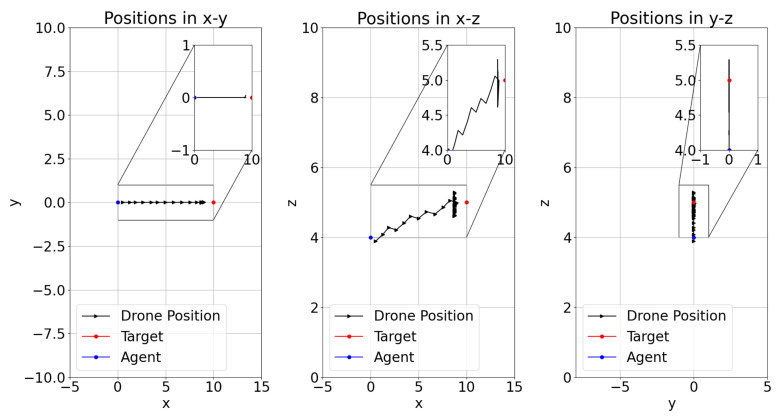
Model-2 Training Episode 3560 Drone Position.

**Figure 15:**
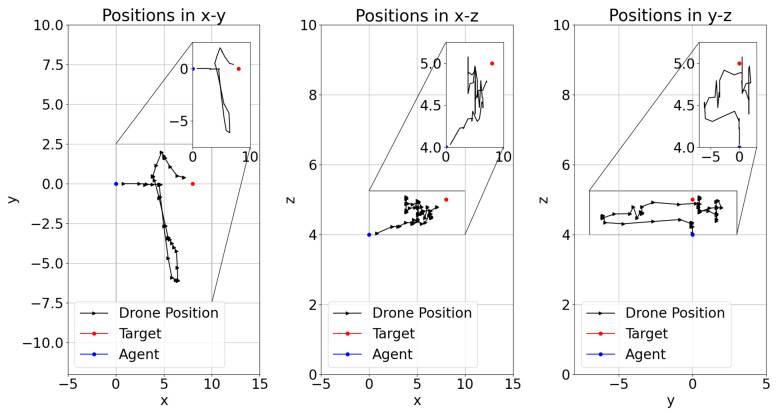
Model-1 Training Episode 2285 Drone Position.

**Figure 16:**
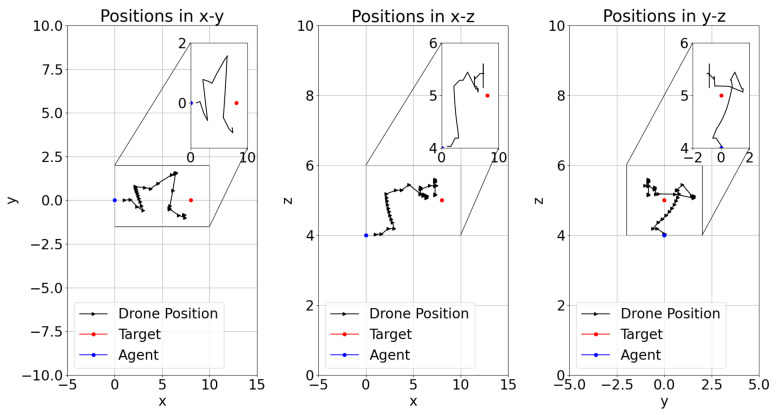
Model-8 Training Episode 2666 Drone Position.

The position of the learner drone, the agent, in episodes during the training of DRL models have shown different flight paths in order to catch the target in a 3D space. Figure 14, Figure 15 and Figure 16 present the positions during training in episodes 3560 for model-2, 2285 for model-1, and 2666 for model-8. Previously, model-1 and model-2 have been chosen as the best models. It is found that the learner drone can use different actions to catch the drone in different episodes and different models. For instance, model-2 takes 58 steps to catch the target and the positions are shown in Figure 14. The learner drone moves up and goes forward without changing position in the y-direction. Figure 15 shows that model-1 can use 102 action steps to catch the target. Firstly, the learner drone moves up and goes forward. After the learner drone passes the target, it starts going up and down to search for the target and finally it catches it. Moreover, model-1 has shown an interesting approach in that the learner drone spends time in the y-direction such as going left and right and going up and down at the same time. However, although model-8 is declared as one of the worst models, there are also successful episodes in which the learner drone catches the target. For example, the learner drone position is presented in Figure 16. In this figure, it is seen that the learner drone spends a lot of time to find the target and uses different kinds of actions including going forward and backwards. The episode in this approach takes 84 steps to catch the target. In counter-drone systems, catching the target as soon as possible is expected. Otherwise, the target can be lost in a short time.

### Deep Q-Learning from Demonstrations (DQfD)

As described earlier, reinforcement learning is an AI method in which the agent can learn from trial-and-error experiences by interacting in an environment. However, learning from scratch can be time consuming in some real-world applications such as counter-drone systems in a 3D space. Experiences from an expert can be used to accelerate the learning speed and the agent can learn in an efficient way. For instance, an AI method called imitation learning has been used to teach an agent to mimic the behavior of an expert. In imitation learning, the labeled data are used as an input and the agent imitates the actions from the recorded data. However, the data are limited to the expert data. On the other hand, a deep reinforcement learning algorithm called Deep Q-learning from Demonstrations [33] is introduced to combine imitation learning and reinforcement learning. In DQfD the agent continues learning by sampling from both its self-generated data as well as the demonstration data.

The main purpose of this algorithm is to remove the limitations of the applicability of DRL to real-world tasks where the agent must learn in the environment. DQfD provides the agent with data from previous control of the system. Thanks to a prioritized experience replay mechanism, DQfD can access the demonstration data to accelerate the learning process even if the agent has a small amount of demonstration data. Researchers showed that DQfD can perform better and learn to out-perform the best demonstration given in 14 of 42 games. In addition, DQfD has already achieved state-of-the-art results on 11 Atari games.

The DQfD algorithm has two phases of training. Firstly, DQfD pretrains on the expert demonstration data using a combination of temporal difference (TD): 1-step TD, n-step TD, supervised, and regularization losses. The TD loss enables the algorithm to learn a self-consistent value function from which it can continue learning with RL. In addition, the supervised loss is used to learn to imitate the demonstrator. After pre-training, the agent starts interacting with the domain with its learned policy. The agent updates its network with a mix of demonstration and self-generated data. The combination of demonstration data and self-generated data is automatically controlled by a prioritized-replay mechanism. The overall loss is presented in Equation (6). In this equation, in addition to TD losses, a margin classification loss $J_{E}$ [34] and L2 regularization loss $J_{L2}$ are implemented. λ parameter is added to control the weighting between the losses. L2 regularization loss is applied to the weights and biases of the network to help prevent it from over-fitting on the relatively small demonstration dataset. A margin classification loss is added to make the greedy policy induced by the value function imitate the demonstrator by forcing the values of the other actions to be at least a margin lower than the value of the demonstrator’s action. After pre-training, the agent starts interacting with the environment, collecting self-generated data and adding it to the replay buffer until it is full, but the demonstration data are never over-written.

(6) $$J\left(Q\right)=J_{DQ}\left(Q\right)+\lambda_{1}J_{n}\left(Q\right)+\lambda_{2}J_{E}\left(Q\right)+\lambda_{3}J_{L2}\left(Q\right)$$

(7) $$J_{E}\left(Q\right)=\max_{a\in A}\left[Q\left(s,a\right)+lQ\left(a_{E},a\right)-Q\left(s,a_{E}\right)\right]$$

where $a_{E}$ is the action the expert demonstrator took in state s and $l(a_{E},a)$ is the margin function.

In this study, a previously trained model, model-2, one of the best models presented in Section 4.1, is used to demonstrate for the DQfD algorithm. After training, the model obtained is tested in the environment. The results showed that the demonstration data help with faster learning. However, there are limitations in some cases such as the complexity of the environment with obstacles, geo/fences and learner drone crashes.

**Figure 17:**
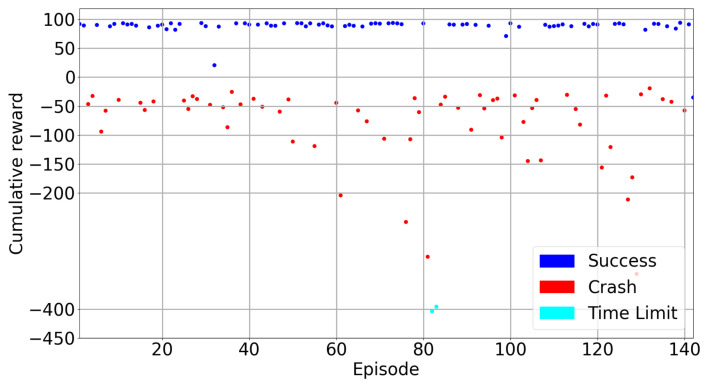
DQfD Train Results.

**Figure 18:**
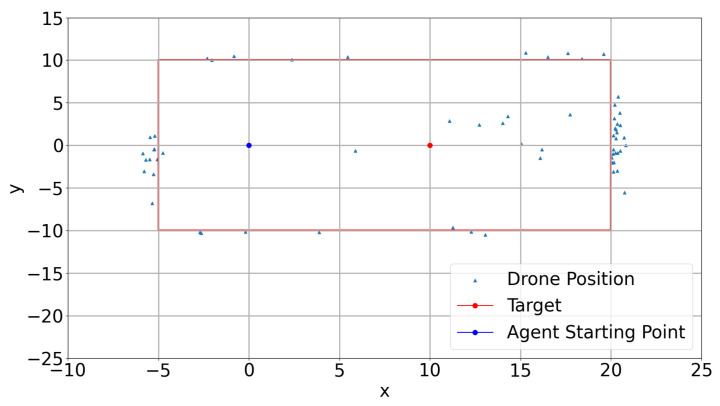
DQfD Crash Positions.

**Figure 19:**
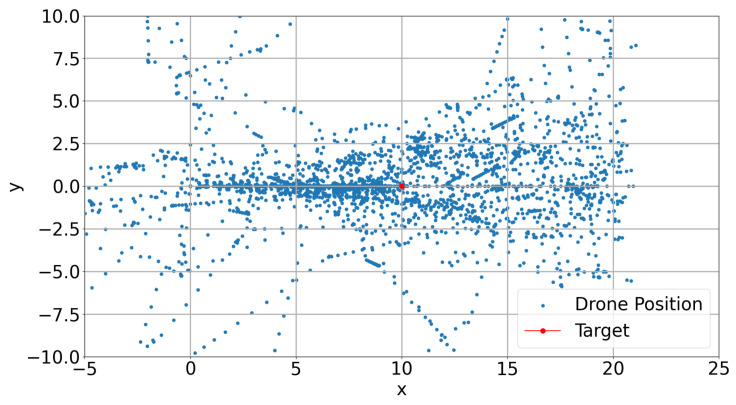
DQfD Drone Positions.

**Figure 20:**
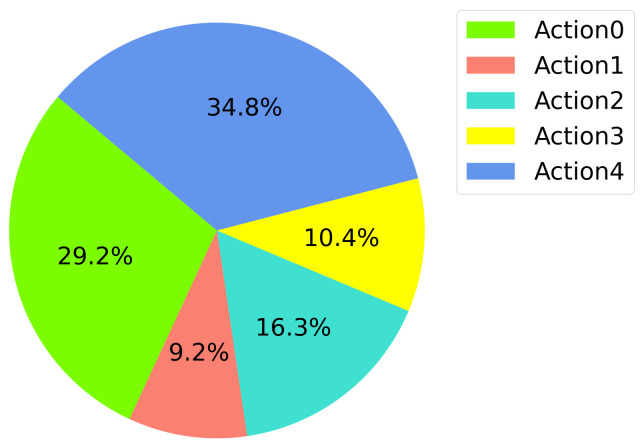
DQfD Action Frequency.

Training results for the DQfD model are shown in Figure 17, Figure 18, Figure 19 and Figure 20. It is seen in Figure 17 that the DQfD model is trained with only 140 episodes. This is less than the other models presented in this paper. However, the learning process is faster considering the time spent for training. There are also few crashed episodes on geo-fences as shown in Figure 18. Crashed positions can also be seen in Figure 19 where the learner drone position is presented. The learner drone focuses on the target and mostly spends time moving towards the target drone position. Considering the action frequency plot shown in Figure 20, moving forward (Action0) and going up (action4) are the expected actions in the environment.

**Figure 21:**
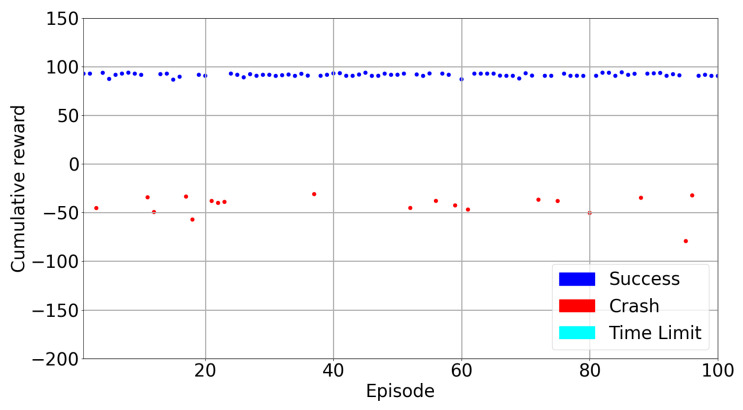
DQfD Model Test Results.

Table 6 presents the training and test rewards, success rates and average steps during training and tests. Training average cumulative rewards in the DQfD model is very low but it is higher in test results. However, almost 20% of the episodes in the test crashed because of geo-fences. The test results are presented in Figure 21. In general, the rewards in the test remain between 90–100 and look stable. The episodes which are not successful have rewards which are mostly around −50 and there is no time limit in this case. These results show that DQfD can be utilized to train the drone in a short time to save time and resources for countering a drone in a 3D space which is a challenging task compared to a 2D space counter-drone solution. However, the DQfD algorithm for a counter-drone system needs improvement to be used in real-world applications. To achieve the best results, a hyper parameter search can be beneficial.

## 6. Conclusions

Counter-drone systems to fight against unknown drones can benefit from artificial intelligence methods. In this paper, an artificial intelligence method called deep reinforcement learning is demonstrated. Countering a drone in a 3D space is a very challenging task compared to countering a drone in a 2D space, and it can be unstable even if it is trained over a long time to catch a drone. The main contributions of this paper have been the usage of a DQN algorithm with dueling network architecture and prioritized experience replay to catch another drone in a 3D space in an environment provided by an Airsim simulator. In addition, experiences from previous training are also transferred before starting a new training by pre-processing the previous experiences and eliminating those considered as bad experiences. The results show that drone learning progress has increased dramatically. Crashes on geo-fences and obstacles in the environment are reduced. The drone detection model running in the background is also implemented in most of the models, and one of the best models is obtained by using the drone detection model running in the background. The neighborhood environment in the simulator requires more intelligent exploration because of different kind of obstacles such as trees, cars, overhead cables in the street, houses, etc. which the drone can crash on. For this reason, another deep reinforcement algorithm, deep q-learning from demonstrations, is also implemented for a counter-drone solution and the training and test results are compared with the other models presented in this study. In this model, demonstration data play an important role to achieve higher rewards during training. The main advantage of this model is that even after pre-training, the DQfD algorithm allows the demonstration data to be used during the training. However, the results are slightly better in terms of the average steps the learner drone takes during the test, but the average cumulative rewards are well below compared to the models presented. Thus, the actions which the demonstrator has taken are not easy to classify, and therefore the differences between the demonstrator and the agent data become more important. In future work, the plan is to improve DQfD for the usage of a counter-drone system by using the human demonstrator. Humans can use a different policy that a learner drone would learn from training, and this information can be hidden in learner drone state representation. Deep reinforcement learning algorithms are developing and there will be challenges in the future. The identification of drones with the help of drone detection systems is a very important part of counter-drone solutions since the state of the target drone is the main part of the deep reinforcement learning algorithm. The correct actions to be taken by the learner drone are also crucial for the counter-drone system since this directly affects the time to interact with the unwanted drone.

## Figures and Tables

**Table 1:** Actions.

Action	Movement
0	2 m/s in +x direction
1	30 deg yaw left
2	25 deg yaw right
3	0.25 m/s in +z direction
4	0.25 m/s in −z direction

**Table 2:** Rewards.

Reward	The Reason
+100	Target Caught
−1 + ΔDistance	Episode steps between 0–50
−2 + ΔDistance	Episode steps between 50–100
−3 + ΔDistance	Episode steps between 100–150
−4 + ΔDistance	Episode steps between 150–200

**Table 3:** Setup DRL Models.

Models	Target Location in X Direction	Target Location in Z Direction	Teleportation and Random Heading	Transfer Learning	Dueling Network Architecture	Image State	Scalar States	Annealed Steps	Training Steps	Drone Detection
Model-1	8	−5	NO	NO	YES	(256,144)	$d_{x}d_{y}d_{z}d_{t}$	15,000	<50,000	NO
Model-2	10	−5	NO	YES	YES	(84,84)	$d_{x}d_{y}d_{z}d_{t}$	20,000	>50,000	YES
Model-3	8	−5	NO	NO	YES	(84,84)	$d_{x}d_{y}d_{z}d_{t}$	15,000	<50,000	YES
Model-4	8	−5	NO	NO	YES	(84,84)	$d_{x}d_{y}d_{z}d_{t}$	20,000	>50,000	YES
Model-5	10	−5	NO	NO	YES	(84,84)	$d_{x}d_{y}d_{z}d_{t}$	15,000	<50,000	YES
Model-6	8	−5	NO	NO	NO	(84,84)	$d_{x}d_{y}d_{z}d_{t}$	15,000	<50,000	YES
Model-7	8	−5	YES	NO	NO	(84,84)	$d_{x}d_{y}d_{z}d_{t}$	100,000	<50,000	YES
Model-8	8	−5	YES (Random Heading)	NO	NO	(84,84)	$d_{x}d_{y}d_{z}d_{t}$	50,000	>50,000	YES
Model-9	8	−5	NO	NO	NO	(84,84)	$d_{x}d_{y}d_{z}d_{t}$	15,000	<50,000	YES

**Table 4:** DRL Models Training Rewards Statistics.

Models	Average Cumulative Reward	Max. Cumulative Reward	Min. Cumulative Reward	Success Rates
Model-1	83.11	94.48	−429.27	95%
Model-2	83.82	94.12	−420.99	98%
Model-3	−3.73	97.33	−426.16	47%
Model-4	65.91	98.09	−427.04	88%
Model-5	65.24	96.16	−424.30	88%
Model-6	64.49	98.21	−416.40	83%
Model-7	−29.40	96.25	−252.38	5%
Model-8	71.80	97.16	−421.06	86%
Model-9	72.08	97.14	−418.69	85%

**Table 5:** DRL Models Test Statistics.

Models	Average Cumulative Reward	Max. Cumulative Reward	Min. Cumulative Reward	Success Rates	Average Steps
Model-1	90.86	91.12	90.69	100%	8.98
Model-2	89.38	92.93	82.93	100%	11.92
Model-7	−87.55	73.78	−408.58	3%	56.97
Model-8	65.73	95.06	−220.29	92%	32.01

**Table 6:** DQfD Model Train and Test Statistics.

DQfD Models	Average Cumulative Reward	Max. Cumulative Reward	Min. Cumulative Reward	Success Rates	Average Steps
DQfD Model Train	7.95	93.67	−403.94	55%	35.21
DQfD Model Test	65.96	94.07	−79.29	81%	16.82

**Figure A2:**
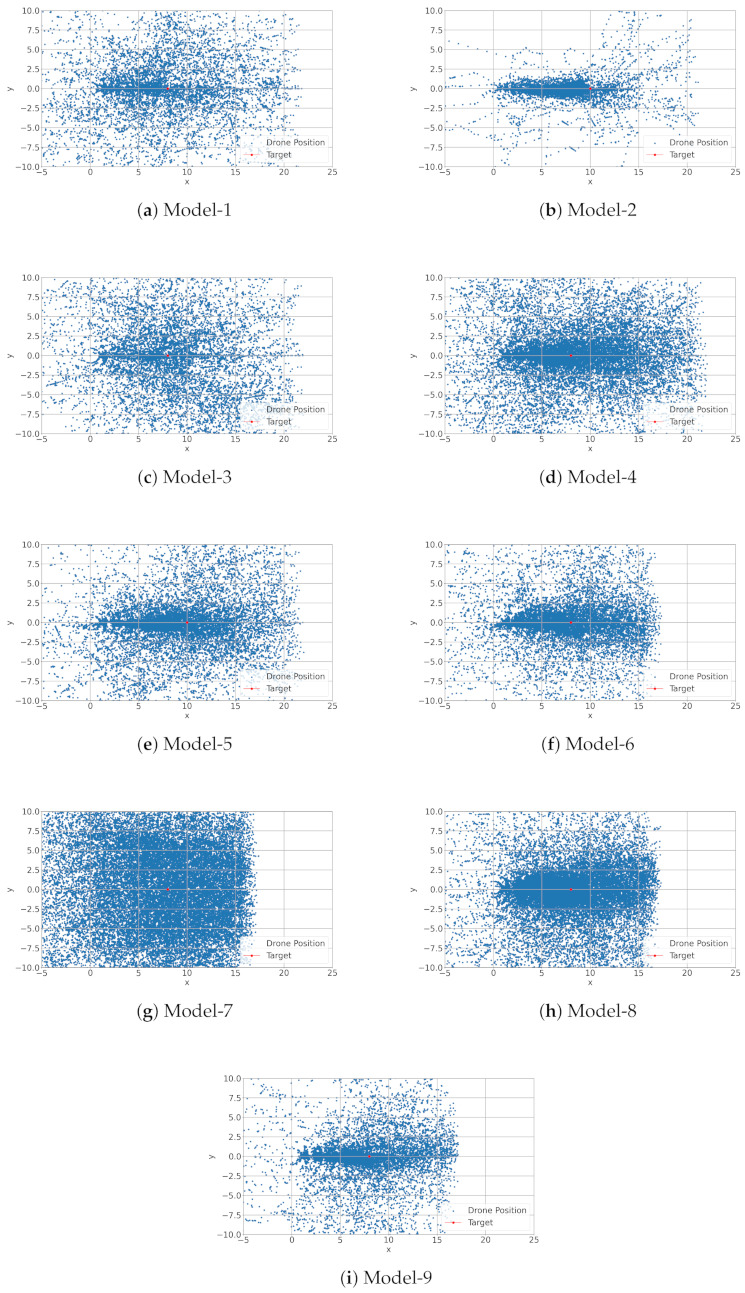
Drone Positions.

## Abbreviations

The following abbreviations are used in this manuscript:

UAV	Unmanned Aerial Vehicle
AI	Artificial Intelligence
DRL	Deep Reinforcement Learning
RL	Reinforcement Learning
TL	Transfer Learning
NED	North East Down
BVLOS	Beyond Visual Line of Sight
DDQN	Double Deep Q-network
PER	Prioritized Experience Replay
DQfD	Deep Q-learning from Demonstrations
CNN	Convolutional Neural Network
TD	Temporal Difference
API	Application Programming Interface

## Appendix A. Results

### Appendix A.1. Training Results

**Table A1:** Hyperparametres of the training.

Hyperparameter	Value	Observations
Training steps	75,000	Changes in different scenarios (50,000–75,000)
Annealing length	15,000	Changes in different scenarios (15,000–45,000)
Annealing interval ϵ	[1–0.1]	Linear Annealed Policy (can be [0.1–0.01])
Steps to warm-up	180	Number of random steps to take before learning begins
Prioritized experience replay, memory limit	100,000	
Prioritized experience replay, alpha	0.6	Decides how much prioritization is used
Prioritized experience replay, beta		Decides how much we should compensate for the non-uniform probabilities
Prioritized experience replay, start-beta	0.4	
Prioritized experience replay, end-beta	0.4	
Pretraining steps	1000	Length of ’pretraining’
Large margin	0.8	Constant value
$Lam_{2}$	1	Imitation loss coefficient
Dueling type	’avg’	A type of dueling architecture
Target model update τ	0.001	Frequency of the target network update
Discount factor γ	0.99	The discount factor of future rewards in the Q function
Learning rate α	0.00025	Adam optimizer [35]

## References

[1] Anti-Drone Market “To Be Worth USD1.5 Billion” by 2023—New Report. https://www.unmannedairspace.info/utm-and-c-uas-market-analysis/anti-drone-market-to-be-worth-usd1-5-billion-by-2023-new-report/.

[2] Drones by the Numbers. https://www.faa.gov/uas/resources/by_the_numbers/.

[3] Chiper F.L., Martian A., Vladeanu C., Marghescu I., Craciunescu R., Fratu O. (2022). Drone Detection and Defense Systems: Survey and a Software-Defined Radio-Based Solution. Sensors.

[4] Drozdowicz J., Wielgo M., Samczynski P., Kulpa K., Krzonkalla J., Mordzonek M., Bryl M., Jakielaszek Z. 35 GHz FMCW drone detection system. Proceedings of the 2016 17th International Radar Symposium (IRS).

[5] Semkin V., Yin M., Hu Y., Mezzavilla M., Rangan S. Drone detection and classification based on radar cross section signatures. Proceedings of the 2020 International Symposium on Antennas and Propagation (ISAP).

[6] Bernardini A., Mangiatordi F., Pallotti E., Capodiferro L. (2017). Drone detection by acoustic signature identification. Electron. Imaging.

[7] Mezei J., Fiaska V., Molnár A. Drone sound detection. Proceedings of the 2015 16th IEEE International Symposium on Computational Intelligence and Informatics (CINTI).

[8] Nguyen P., Ravindranatha M., Nguyen A., Han R., Vu T. Investigating cost-effective RF-based detection of drones. Proceedings of the 2nd Workshop on Micro Aerial Vehicle Networks, Systems, and Applications for Civilian Use.

[9] Opromolla R., Fasano G., Accardo D. (2018). A vision-based approach to UAV detection and tracking in cooperative applications. Sensors.

[10] De Haag M.U., Bartone C.G., Braasch M.S. Flight-test evaluation of small form-factor LiDAR and radar sensors for sUAS detect-and-avoid applications. Proceedings of the 2016 IEEE/AIAA 35th Digital Avionics Systems Conference (DASC).

[11] Çetin E., Barrado C., Pastor E. (2021). Improving real-time drone detection for counter-drone systems. Aeronaut. J..

[12] Aker C., Kalkan S. Using deep networks for drone detection. Proceedings of the 2017 14th IEEE International Conference on Advanced Video and Signal Based Surveillance (AVSS).

[13] Lykou G., Moustakas D., Gritzalis D. (2020). Defending airports from UAS: A survey on cyber-attacks and counter-drone sensing technologies. Sensors.

[14] Watkins L., Sartalamacchia S., Bradt R., Dhareshwar K., Bagga H., Robinson W.H., Rubin A. Defending against consumer drone privacy attacks: A blueprint for a counter autonomous drone tool. Proceedings of the Decentralized IoT Systems and Security (DISS) Workshop 2020.

[15] Barišic A., Petric F., Bogdan S. (2022). Brain over Brawn: Using a Stereo Camera to Detect, Track, and Intercept a Faster UAV by Reconstructing the Intruder’s Trajectory. arXiv.

[16] Bertoin D., Gauffriau A., Grasset D., Gupta J.S. Autonomous drone interception with Deep Reinforcement Learning. Proceedings of the ATT’22: Workshop Agents in Traffic and Transportation.

[17] Shim D.H. (2021). Development of an Anti-Drone System Using a Deep Reinforcement Learning Algorithm. Ph.D. Thesis.

[18] Akhloufi M.A., Arola S., Bonnet A. (2019). Drones Chasing Drones: Reinforcement Learning and Deep Search Area Proposal. Drones.

[19] He L., Aouf N., Whidborne J.F., Song B. (2020). Deep reinforcement learning based local planner for UAV obstacle avoidance using demonstration data. arXiv.

[20] Çetin E., Barrado C., Pastor E. (2020). Counter a Drone in a Complex Neighborhood Area by Deep Reinforcement Learning. Sensors.

[21] Tan M., Pang R., Le Q.V. Efficientdet: Scalable and efficient object detection. Proceedings of the IEEE/CVF Conference on Computer Vision and Pattern Recognition.

[22] Brockman G., Cheung V., Pettersson L., Schneider J., Schulman J., Tang J., Zaremba W. (2016). OpenAI Gym. arXiv.

[23] Abadi M., Agarwal A., Barham P., Brevdo E., Chen Z., Citro C., Corrado G.S., Davis A., Dean J., Devin M. (2015). TensorFlow: Large-Scale Machine Learning on Heterogeneous Systems. arXiv.

[24] Plappert M. (2016). keras-rl. https://github.com/keras-rl/keras-rl.

[25] Shah S., Dey D., Lovett C., Kapoor A. (2017). AirSim: High-Fidelity Visual and Physical Simulation for Autonomous Vehicles. arXiv.

[26] Sutton R.S., Barto A.G. (1998). Reinforcement Learning: An Introduction.

[27] Mnih V., Kavukcuoglu K., Silver D., Rusu A.A., Veness J., Bellemare M.G., Graves A., Riedmiller M., Fidjeland A.K., Ostrovski G. (2015). Human-level control through deep reinforcement learning. Nature.

[28] Van Hasselt H., Guez A., Silver D. (2016). Deep Reinforcement Learning with Double Q-Learning. arXiv.

[29] Mnih V., Kavukcuoglu K., Silver D., Graves A., Antonoglou I., Wierstra D., Riedmiller M.A. (2013). Playing Atari with Deep Reinforcement Learning. arXiv.

[30] Wang Z., Schaul T., Hessel M., Hasselt H., Lanctot M., Freitas N. Dueling network architectures for deep reinforcement learning. Proceedings of the 33rd International Conference on International Conference on Machine Learning.

[31] Schaul T., Quan J., Antonoglou I., Silver D. (2015). Prioritized experience replay. arXiv.

[32] Unreal Engine 4. https://www.unrealengine.com/en-US/what-is-unreal-engine-4.

[33] Hester T., Vecerik M., Pietquin O., Lanctot M., Schaul T., Piot B., Horgan D., Quan J., Sendonaris A., Osband I. Deep q-learning from demonstrations. Proceedings of the Thirty-Second AAAI Conference on Artificial Intelligence and Thirtieth Innovative Applications of Artificial Intelligence Conference and Eighth AAAI Symposium on Educational Advances in Artificial Intelligence.

[34] Piot B., Geist M., Pietquin O. Boosted and reward-regularized classification for apprenticeship learning. Proceedings of the 2014 International Conference on Autonomous Agents and Multi-Agent Systems.

[35] Kingma D.P., Ba J.L. Adam: Amethod for stochastic optimization. Proceedings of the 3rd International Conference for Learning Representations.

